# Expression, Purification, Crystallization, and Enzyme Assays of Fumarylacetoacetate Hydrolase Domain-Containing Proteins

**DOI:** 10.3791/59729

**Published:** 2019-06-20

**Authors:** Alexander K. H. Weiss, Max Holzknecht, Elia Cappuccio, Ilaria Dorigatti, Karin Kreidl, Andreas Naschberger, Bernhard Rupp, Hubert Gstach, Pidder Jansen-Dürr

**Affiliations:** 1 Research Institute for Biomedical Aging Research, University of Innsbruck Austria; 2 Center for Molecular Biosciences Innsbruck (CMBI), University of Innsbruck Austria; 3 Division of Genetic Epidemiology, Medical University of Innsbruck Austria; 4 Faculty of Chemistry, Department of Organic Chemistry, University of Vienna Austria

**Keywords:** Biochemistry, Issue 148, FAH, FAHD, FAHD1, ODx, ApH, hydrolase, decarboxylase, oxaloacetate, acetylpyruvate, fumarylpyruvate, acylpyruvate, oxalate, pyruvate, MiDAS

## Abstract

Fumarylacetoacetate hydrolase (FAH) domain-containing proteins (FAHD) are identified members of the FAH superfamily in eukaryotes. Enzymes of this superfamily generally display multi-functionality, involving mainly hydrolase and decarboxylase mechanisms. This article presents a series of consecutive methods for the expression and purification of FAHD proteins, mainly FAHD protein 1 (FAHD1) orthologues among species (human, mouse, nematodes, plants, etc.). Covered methods are protein expression in *E. coli*, affinity chromatography, ion exchange chromatography, preparative and analytical gel filtration, crystallization, X-ray diffraction, and photometric assays. Concentrated protein of high levels of purity (>98%) may be employed for crystallization or antibody production. Proteins of similar or lower quality may be employed in enzyme assays or used as antigens in detection systems (Western-Blot, ELISA). In the discussion of this work, the identified enzymatic mechanisms of FAHD1 are outlined to describe its hydrolase and decarboxylase bi-functionality in more detail.

## Introduction

The fumarylacetoacetate hydrolase (FAH)^1,2^ superfamily of enzymes describes a group of enzymes that share the highly conserved catalytic FAH domain^3,4,5,6,7,8,9,10^. Despite their common catalytic center, these enzymes are multi-functional, and most are found in prokaryotes, where they are used to break down compounds retrieved from complex carbon sources^3^. Only three members of this family were identified in eukaryotes so far: the name giving FAH^2^, as well as FAH domain-containing protein 1 (FAHD1)^11,12,13,14,15^ and FAH domain-containing protein 2 (FAHD2). Depletion of FAHD1 has been associated with impaired mitochondrial respiration^13,16^ and associated with a reversible type of cellular senescence phenotype^14^ that is linked to intermediate potential shortcomings in the electron transport system. Human FAHD1 and its orthologues in model systems (mouse, nematode, cancer cell lines, plants, etc.), as well as selected point mutation variants, have become druggable targets of potential interest. For this research, recombinant protein at high levels of purity, as well as information on catalytic mechanisms guided by crystal structures and selective antibodies are vital.

This manuscript describes methods for FAHD protein expression in *E. coli*, affinity chromatography, ion exchange chromatography, ammonium sulfate precipitation, preparative and analytical gel filtration, crystallization, X-ray diffraction, and photometric assays. The purpose of the methods and protocols described here is to provide guidance for scientists working in diverse fields such as bacteriology, plant biology, as well as animal and human studies, to characterize members of the FAH superfamily, including uncharacterized superfamily members should they become relevant in a particular field. The protocols described here may provide valuable support for projects aiming to characterize other prokaryotic or eukaryotic FAH superfamily members.

The rationale behind the methods described here is the fact that for characterization of poorly described proteins (in particular, metabolic enzymes of unknown physiological relevance), the approach to start with purified recombinant proteins allows the development of invaluable, high-quality research tools such as in vitro active enzyme preparations, high-quality antibodies, and potent and specific pharmacological inhibitors for selected enzymes. The described methods require fast protein liquid chromatography (FPLC) and X-ray crystallography. Alternative methods (e.g., to express protein without chemical induction, or to display protein purification by centrifugation after heat treatment followed by desalting and size exclusion chromatography), may be found elsewhere^17^. While a broader spectrum of methods is available for the expression and purification of FAH superfamily enzymes^2,7,9,17,18^, this work focuses on the expression and purification of FAHD proteins in particular.

In the discussion section of this manuscript, the catalytic mechanisms identified for the FAHD1 protein (hydrolase, decarboxylase)^15^ are described in more detail, in order to demonstrate the chemical character of the catalyzed reactions. The data obtained based on previous work^7,15,18^ (PDB: 6FOG, PDB:6FOH) imply a third activity of the enzyme as keto-enol isomerase.

## Protocol

### 1 Expression of FAHD proteins in competent *E. coli*

**Figure 1:**
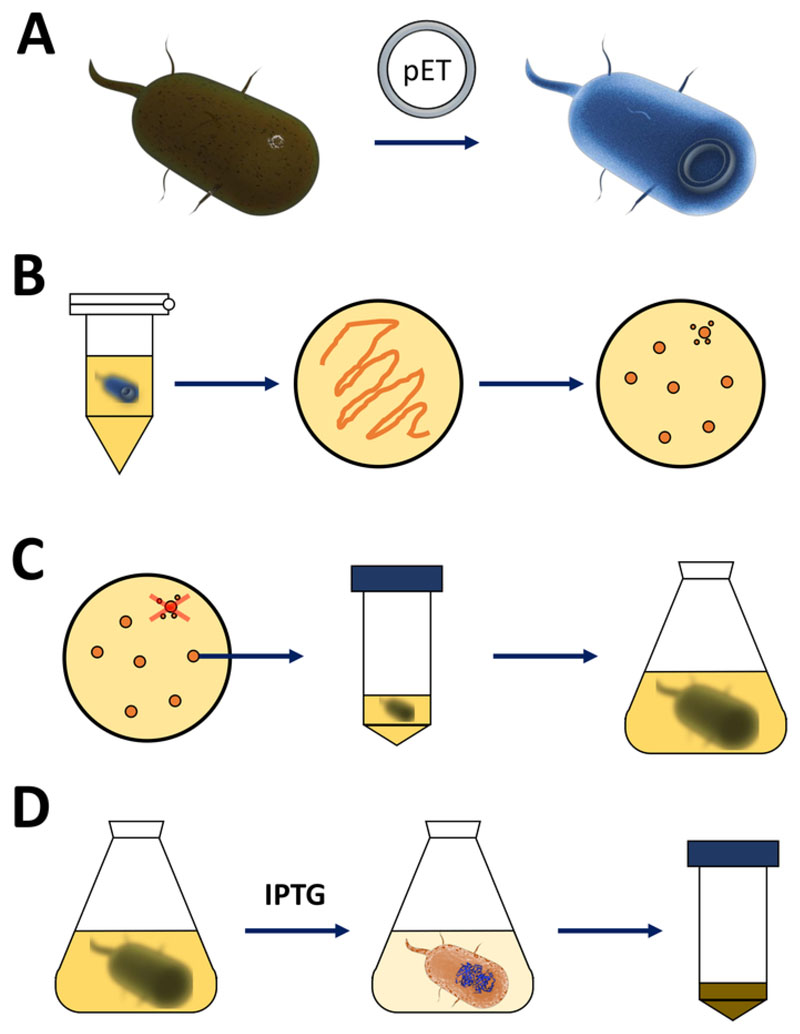
Amplification of competent *E. coli* and induction of protein expression. **(A)** Insertion of the *pET* vector into competent BL21(DE3) *pLysS E. coli* bacteria, described in section 1. **(B)** Heat shock protocol and plating of the *pET* transformed *E. coli* bacteria, described in step 1 of the protocol. Transformed bacteria are plated on LB agar plates with antibiotics for selection. **(C)** Amplification of *pET* transformed *E. coli* bacteria, described in section 1. Colonies are picked from an LB agar plate and amplified in nourishing medium (LB or NZCYM) until the bacterial density reached the empirical threshold of 0.4. **(D)** Induction of protein expression via the DE3-IPTG-*pET* system, described in section 1 and sketched in Figure 2. Protein production is started by the application of the chemical IPTG. At the end of section 1, the bacterial pellet containing the protein is harvested. Please click here to view a larger version of this figure.

**Figure 2:**
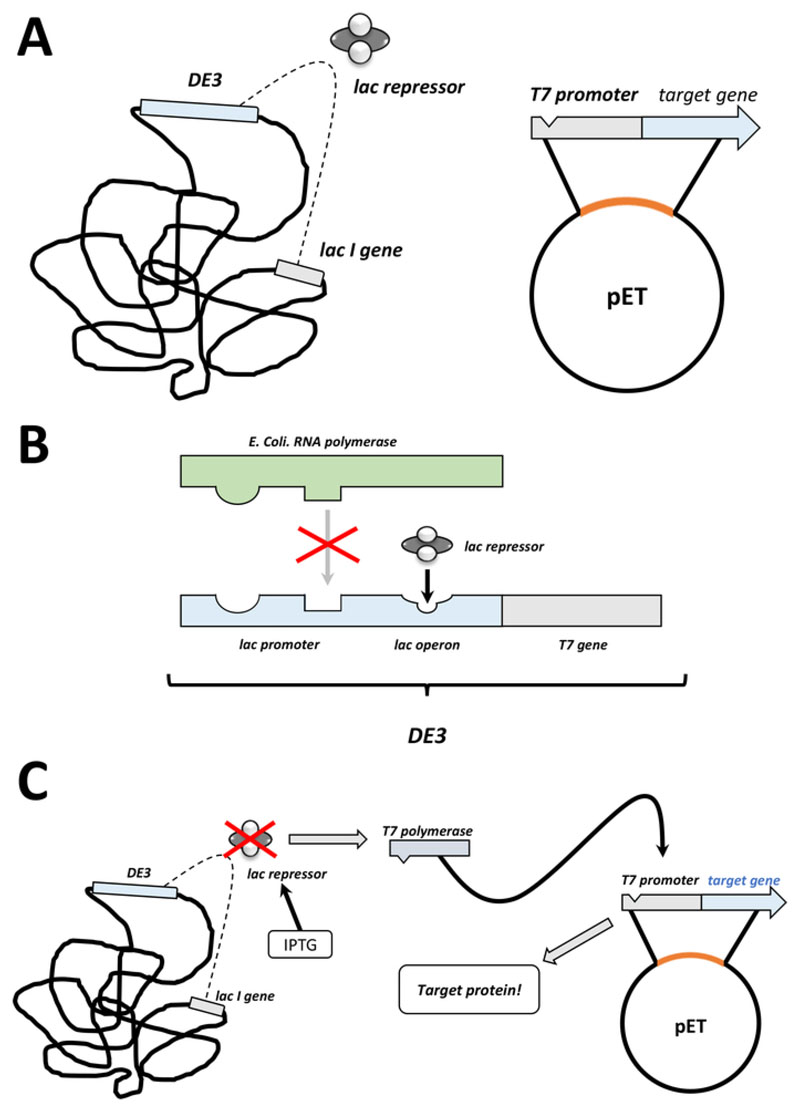
The DE3 cassette/pET vector dual system explained. **(A)** The sketched genome of *pET* vector transformed BL21(DE3) *pLysS E. coli* bacteria. The native bacterial genome carries a DE3 cassette (see panel B), as well as a lac gene that constantly expresses lac repressor units. The non-native *pET* vector carries the protein gene inserted between a *T7* polymerase promoter and terminator sequence. More details in panel B. **(B)** The DE3 cassette of the native bacterial genome encodes the information for *T7* polymerase in terms of an *E. coli* RNA polymerase operon. This protein, however, is not expressed because the lac repressor unit prevents the RNA polymerase protein from binding. Hence no T7 polymerase is expressed and no exogenous protein is expressed. **(C)** Application of the chemical IPTG (Table of Materials) distorts the structure of lac repressor units and prevents them from binding to the DE3 cassette. As a result, RNA polymerase can now bind to the cassette, for which T7 polymerase is expressed, as is exogenous protein eventually. Please click here to view a larger version of this figure.

1. **Transformation of *E. Coli* with vectors for expression of FAHD protein**
  NOTE: The steps discussed in the following section are summarized in the sketch in Figure 1A,B. The same protocol applies for any FAHD protein, including point-mutant variants. Such variants may be obtained via site-directed mutagenesis and PCR techniques^19^ (such as two-sided SOE PCR^20^) from wild-type cDNA.
  1. Obtain competent BL21(DE3) *pLysS E. coli* bacteria and a pET expression vector (see Table of Materials). Preferably choose a pET vector that also encodes an *N*-terminal His-tag or related capture tag for convenience to simplify the following purification steps.
  2. Obtain cDNA of the FAHD protein of choice and insert it into the active cloning site of the pET expression vector, in between the T7 promoter and T7 terminator sites, respectively.
  3. After successful plasmid amplification and verification [via sequencing by a commercial supplier (T7 primers may be used with the pET system for convenience: T7 promoter, forward primer: TAATACGACTCACTATAGGG; T7 terminator, reverse primer: GCTAGTTATTGCTCAGCGG)], insert 5–10 ng of plasmid into 100 μL of competent BL21(DE3) *pLysS E. coli* bacteria on ice. Do not aspirate up and down, but slightly tap the tube with in order to mix the content.
  4. Keep the bacteria on ice for 30 min, gently tapping the tube every few min.
  5. Heat a heating device or water bath to 42 °C (exact). Put the tube containing the bacteria into the apparatus and keep them for 90 s (exact). Put them on ice immediately (Figure 1A).
  6. After 5–10 min on ice, add 600 μL of NCZYM medium (see Table of Materials) and put the tube into a bacteria incubator. Shake the tube at medium speed oriented along the shaking direction at 37 °C for 1 h.
  7. Plate 200 μL of the bacterial culture on a 10 cm LB-agar plate (see Table of Materials), containing selection antibiotics of choice [e.g., one specific for the BL21(DE3) *pLysS* resistance (chloramphenicol), and one for the resistance encoded on the *pET* vector (kanamycin or ampicillin, Figure 1B)].
  8. Culture the bacteria on the LB-agar plate in a bacterial incubator at 37 °C overnight.
2. **Expression of FAHD proteins by IPTG induction**
  NOTE: The first steps discussed in the following section are summarized as a sketch in Figure 1C,D. The *T7* expression system via combination of the bacterial DE3 cassette and *pET* vector system are summarized in Figure 2.
  1. After successful colony formation, pick one single colony (without any satellite colonies) and disperse it in 5 mL of NZCYM or LB medium with antibiotics, selected as before (step 1.1.7). Culture in the bacterial incubator at 37 °C overnight (Figure 1C).
  2. After successful bacterial growth, amplify the bacteria in 250 mL, 500 mL, or 1 L batches of medium, depending on the demand of protein quantity.
    1. Appropriate to the volume, apply antibiotics selected as done in step 1.1.7 and add about 1%–2% of dense bacterial pre-culture (i.e., 2.5–5.0 mL to 250 mL volume of medium, etc.). Take a sample to be used in step 1.2.5 (1 mL or more) and check the optical density (OD) at 600 nm. Culture bacteria in the bacterial incubator at 37 °C for 2–3 h (Figure 1C).
  3. After 2–3 h, draw a sample for photometric analysis. If the OD at 600 nm has reached 0.4, apply 200 μM up to 1 mM isopropyl-β-D-thiogalactopyranosid (IPTG, see Table of Materials).
    NOTE: The actual value is empirical for each FAHD protein or point mutation variant, where 1 mM IPTG is the maximum that should be applied. This induces protein expression (Figure 1D, Figure 2C).
  4. After 3–5 more hours in the bacterial incubator at 37 °C, protein expression is exhausted.
    NOTE: See the discussion section for comments on temperature control. Longer than 5 h of shaking after induction is not recommended. Take a sample for use in step 1.2.5 (1 mL or more) and check the optical density (OD) at 600 nm.
    1. Harvest the bacterial pellet *via* centrifugation at 5,000 x *g* for 5 min. Discard the supernatant and freeze the pellet at -80 °C for longer storage or -20 °C for brief storage (Figure 1D).
  5. Verify induction via the two retrieved photometric samples, that are labelled “-I” (before induction) and “+I” (after induction). After centrifugation and resuspension of the bacterial pellet, analyze the two samples by SDS-PAGE by loading the same amount of total protein.
    NOTE: The “+I” sample should display a strong band associated with the molecular weight of the chosen protein, whereas the “-I” sample should not contain this band. A low induction level is a common problem for production of proteins, yet the level of expressed protein is often sufficient for the following steps. A high induction level is an advantage but is not mandatory.

### 2 Lysis of bacterial pellets and filtration of debris

1. Dependent on whether the chosen protein is *His*-tagged or untagged, select Ni-NTA running buffer (*His*-tagged, see Table of Materials) or ice-cold HIC running buffer (untagged).
2. For each 250 mL of original bacterial suspension, apply 5 mL of the selected buffer to the bacterial pellet (5 mL for 250 mL, 10 mL for 500 mL, etc.). Add 10 μL β-mercaptoethanol (β-ME) per 5 mL of applied buffer. Use a 10 mL Pasteur pipet to mechanically force the pellet into suspension by scratching and pipetting (avoid air bubble formation while pipetting). Eventually transfer all of the suspension into one 50 mL tube.
3. Preferably sonicate (6x for 15 s at medium force) the suspension.
4. Centrifuge for 30 min at high speed (10,000 x *g*) at 4 °C. Filter the supernatant consecutively with filter units (e.g., 0.45 μm, 0.22 μm) on ice.
  NOTE: Depending on the previous centrifugation step, filtration directly through a small filter pore size may be tedious and usually requires pre-filtration through a larger pore size. DNAse may be added for better results.
5. Store the sample on ice and proceed immediately with either section 3 or 4, depending on whether the protein is *His*-tagged or untagged.

### 3 Purification of *His*-tagged FAHD proteins using Ni-NTA affinity chromatography

**Figure 3:**
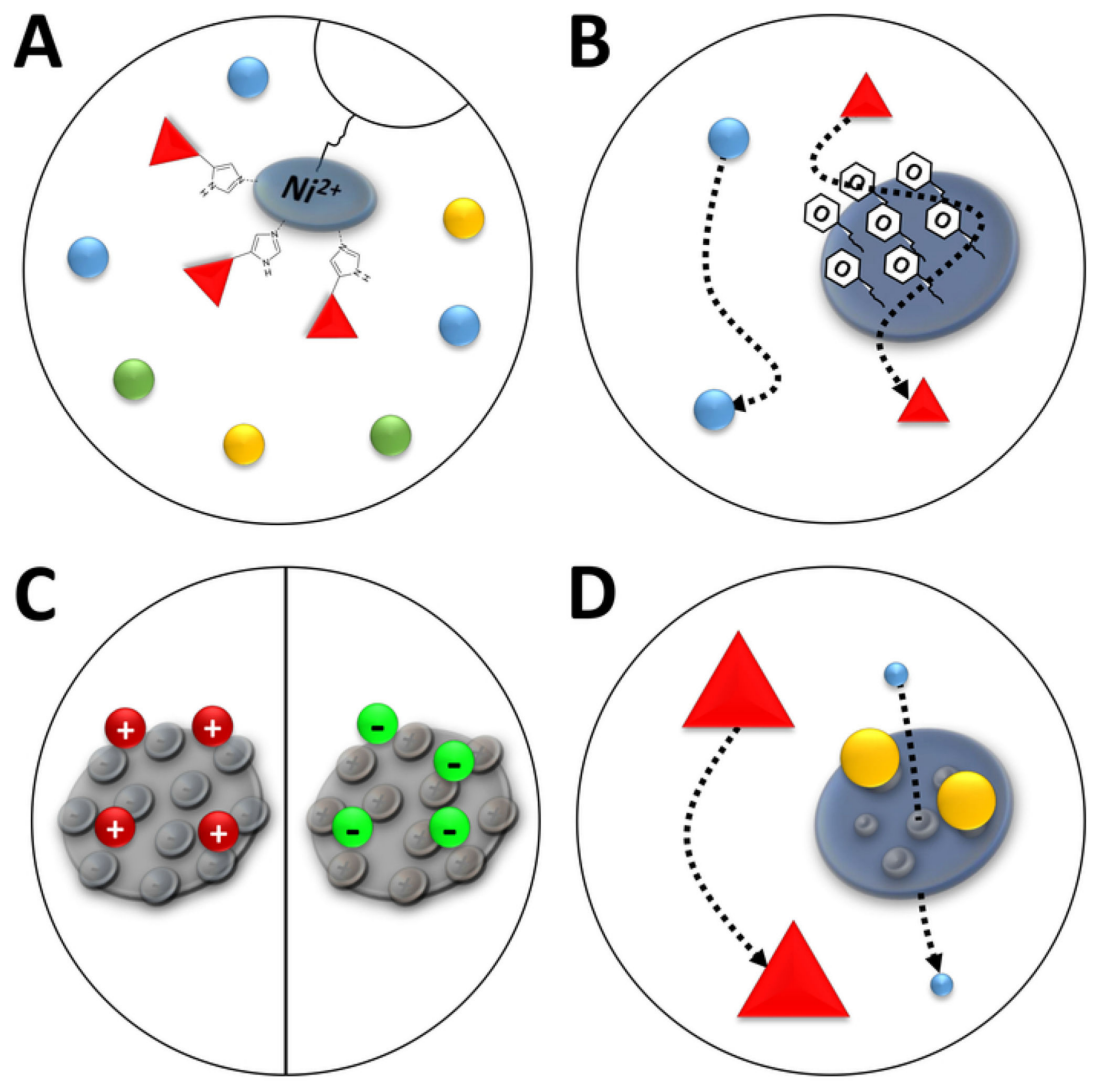
Sketched illustrations of common types of chromatography. **(A)** The resin of a Ni-NTA column. NTA holds bivalent nickel ions that are used in terms of immobilized metal ion affinity chromatography (IMAC). Poly-histidine tags bind preferably to this motif and may be eluted by imidazole. **(B)** The typical coating of silica particles in a phenyl-based hydrophobic interaction chromatography (HIC-phenyl). Hydrophobic proteins interact with the coating material and are delayed in their migration while others are not. **(C)** The typical coating of silica particles in ionic interaction chromatography. Polarized and charged proteins interact with the coating material and are delayed in their migration while others are not. **(D)** The resin of a silica gel in size-exclusion chromatography (SEC). Based on defined pores in the silica material, proteins may be separated by their size (in a first approximation corresponding to their molecular mass). Small proteins permeate the porous column material and are retarded, while large proteins migrate faster around the porous particles. Please click here to view a larger version of this figure.

NOTE: Ni^2+^ ions are bound via nitrilotriacetic acid (NTA) to an agarose resin that is used in affinity chromatography (immobilized metal ion chromatography, IMAC, Figure 3A). Poly-histidine amino acid tags bind strongly to this Ni-chelate, and His-tagged proteins can be separated from the majority of remaining proteins. An alternative to the described preparation of Ni-NTA columns is using prepacked Ni-NTA columns and a FPLC system.

1. Proceed from step 2.5 (i.e., the protein is in Ni-NTA running buffer and filtered by 0.22 μm filter units on ice).
2. Prepare an empty plastic or glass column by washing the empty column and attaching it to a stable retainer. Choose the size of the column depending on the volume of the protein suspension.
3. For each 10 mL of protein suspension, apply 500 μL of Ni-NTA agarose slurry into the column (shake heavily before usage). Apply the slurry slowly and dropwise onto the bottom filter of the column using a pipette. Let the column settle, which takes a few seconds.
4. Fill the column completely with Ni-NTA running buffer, ensuring not to disrupt the agarose resin. Let the buffer run through by gravity. The process may be accelerated up by applying thumb pressure onto the liquid (using a lid or glove and thumb pressure), but take care not to distort the agarose resin.
5. Apply the protein suspension. As before, let the sample run through by gravity. Accelerating this step using thumb pressure is not recommended, as binding of proteins to the column is enhanced if the flow rate is low. Collect the flow-through in a tube (Table of Materials).
6. After the sample has passed through, fill the whole column again with Ni-NTA running buffer. Take care to not disrupt the agarose resin. Let the sample run through by gravity, but in contrast to the previous step, accelerating the process via thumb pressure is recommended, as potential contaminations because of unspecific interactions may be disrupted this way. Collect the washing solution in a tube. Repeat this step.
7. Place a UV-transparent cuvette below the column and apply 1 mL of Ni-NTA elution buffer. Collect the sample without applying any thumb pressure to the resin.
8. Check the optical density (OD) of the sample at 280 nm vs. a blank sample (i.e., Ni-NTA elution buffer). Optimally, the sample displays an OD of greater than 2.5. An OD below 0.5 denotes that no significant amount of protein is in the sample.
  NOTE: As outlined in the discussion section, salt and imidazole concentrations of the elution buffer may have to be adapted for each FAHD protein individually.
9. Repeat steps 3.1.7 and 3.1.8 until the OD falls below 0.5. Pool all samples with higher OD in a tube on ice.
10. Start again with step 3.1.4, using the flow-through from step 3.1.5 as new input for this repetition of step 3.1.5. Repeat this process until the first sample collected in step 3.1.6 displays an OD below 0.5.
  NOTE: As outlined in the troubleshooting part of the discussion section, His-tagged proteins may bind insufficiently to the Ni^2+^-resin. In such cases, repetition of this step or alternative methods (e.g., ion exchange chromatography) are required.
11. Take samples of all intermediate fractions for SDS-PAGE analysis.
12. FAHD proteins in Ni-NTA elution buffer will precipitate upon freezing and thawing. Therefore, dialyze the protein against a different buffer (overnight on ice, using 1 μL of DTT per 100 mL of dialysis buffer). Use low-salt buffer based on which type of ion-exchange chromatography should be performed after this step. Use common cellulose tubing with a typical molecular weight cut-off of 14 kDa (Table of Materials).
13. After overnight dialysis, optionally concentrate the protein using ultra-centrifugation filter units. Perform SDS-PAGE analysis (12.5% running gel, 4% stacking gel) to check for potential loss of protein, insufficient elution, and protein purity in general. If all is fine, proceed to section 5.

### 4 Purification of untagged FAHD proteins via hydrophobic interaction chromatography (HIC)

NOTE: Phenyl-groups on the coating surface of a silica gel in a HIC column for FPLC (Figure 3B) enable the separation of proteins according to hydrophobic character. The described steps should be performed with an FPLC system equipped with a 5 mL of HIC-phenyl column. Columns may be washed with 1 M NaOH to be reused for different proteins. However, columns once used for one type of FAHD protein should be reused for only this type of protein.

1. **Ammonium sulfate (AS) precipitation**
  1. Proceed from step 2.5. The protein is in ice-cold HIC running buffer (Table of Materials).
  2. Assess the volume of the prepared protein solution precisely to the microliter (V_initial_). Slowly and drop-wise add pre-cooled HIC running buffer AS solution, until a 35 volume-% AS saturation is reached: V_AS_ added = V_initial_ * 0.538. Gently stir the solution for 30 min. Centrifuge for 15 min at high speed (≥10,000 x *g*) at 4 °C.
  3. Filter the supernatant using a 0.22 μm filter unit on ice. Optionally, take a sample for SDS-PAGE analysis: dilute 1:4 and heat immediately at 95 °C for 5 min or else the sample will lump. The sample may be frozen at this point (-20 °C) in order to proceed another day.
2. **FPLC using a HIC column**
  1. Setup the FPLC system and equilibrate a 5mL HIC-phenyl column with 5 column volumes (CV) of 20% EtOH (in H_2_O) followed by 5 CV of H_2_O.
  2. Mix 260 mL of HIC running buffer (exact) with 140 mL of HIC running buffer AS (exact). This results in a 35 volume-% AS solution. Check the pH (7.0); this is buffer A. Buffer B is 250 mL of running buffer. Add 1 mM DTT to both buffers A and B, then keep them on ice.
  3. Equilibrate the column with 8 mL of buffer A, 8 mL of buffer B, and 8 mL of buffer A in this sequence. Apply the sample prepared in protocol step 4.1. Wash with buffer A, until the baseline optical absorption at 280 nm reaches 1000–500 mAU.
  4. Apply a mixture of buffers A and B, so that the concentration of AS is 33 % (w/v). Wash with 1 CV, resulting in a plateau in the chromatogram. Set up a gradient of buffer B (up to 100% buffer B over time): 1.5 mL of buffer B in 3.8 min (i.e., 5.7% buffer B with 1% B/mL slope). When the UV signal at 280 nm rises, start collecting the fraction and place it on ice immediately.
  5. In the end, wash the column with buffer B. Take samples of all fractions for SDS-PAGE analysis. Freeze all samples using liquid nitrogen, and store them at -80 °C.
  6. Perform SDS-PAGE (and western blot) analysis, to detect the FAHD protein in the collected fractions. Fractions that contain the protein are pooled and applied to further purification, as outlined in the following protocol steps. Wash the column with H_2_O and 20% EtOH (in H_2_O).

### 5 Purification of FAHD proteins via ion exchange chromatography

NOTE: Molecules with charged functional groups are bound to a silica particle column for FPLC (Figure 3C). This enables the differentiation of proteins according to their ionic character, such as surface charge. The described steps should be performed with an FPLC machine and associated know-how, respectively. The described method is the same for either cationic or anionic exchange chromatography, but the buffers to be used are slightly different.

1. Chose the cationic or anionic exchange chromatography system. This choice is empirical and may vary among FAHD proteins. Optimally, both methods can be used consecutively.
2. Setup the FPLC system and wash the column with 5 CV of 20% EtOH (in H_2_O), followed by 5 CV of H_2_O. Equilibrate the column with 1 CV of low-salt buffer, high-salt buffer, and again low-salt buffer in this sequence.
3. Apply the sample (dialyzed against the correct low salt buffer from step 3.1.11) onto the column. Collect the flow-through. Wash the column for 1 CV with low salt buffer.
4. Setup a gradient elution: 100% high-salt buffer in 30 min at a flow rate of 1 mL/min, or 60 min at a flow rate of 0.5 mL/min. This may be reselected based on an already known FPLC chromatogram, in order to optimize the purification. Collect all peak fractions.
  NOTE: High-salt conditions may vary among FAHD proteins, as outlined in the discussion section.
5. After the gradient has finished, run with high-salt buffer until no more peaks are detected over the range of 1 CV (collect the fractions).
6. Take samples of all collected fractions and perform SDS-PAGE analysis (12.5% running gel, 4% stacking gel). Freeze the individual samples in liquid nitrogen and store them at -80 °C.
7. After SDS-PAGE analysis is complete, pool the samples containing the FAHD protein and discard the others. Optionally, concentrate the protein using ultra-centrifugation filter units.
8. Apply 1 mL of 25% SDS in 0.5 M NaOH (or other detergents) to clean the column. Wash the column with H_2_O and 20% EtOH (in H_2_O).
9. Optionally, repeat section 5 with the alternate column (cationic or anionic exchange chromatography). The protein obtained from this method is sufficiently pure to perform basic activity assays or can be used in screening assays for crystallography. For advanced applications, proceed with section 6.

### 6 Purification of FAHD proteins via size-exclusion chromatography (SEC)

NOTE: Porous particles in a silica gel column for FPLC enable the differentiation of proteins according to molecular size, such as hydrodynamic radius (Figure 3D). The described steps are to be performed with an FPLC system, using SEC columns.

1. Choose an SEC column, dependent on the molecular weights of contaminations still present, as detected via SDS-PAGE and silver staining. The outlined method is suitable for both columns. Wash the column overnight with 400 mL of H_2_O and equilibrate with SEC running buffer. It is recommended to write a program for the FPLC system to automate this step.
2. Add 1 mM DTT to 300 mL of SEC running buffer and put it on ice. This is the running buffer. Apply 60 mL of this buffer to the column.
3. Centrifuge the protein sample (10,000 x *g* for 10 min) to remove any micro-precipitation. Apply the supernatant to the column. It is generally recommended to filter the supernatant before FPLC.
4. Apply the running buffer to the column until all protein is eluted. Collect all peaks in fractions of suitable volume (e.g., 2 mL). Take samples for SDS-PAGE and freeze all fractions using liquid nitrogen. Store the frozen fractions at -80 °C.
5. After SDS-PAGE (and western blot) analysis, collect and pool all fractions containing the FAHD protein. Silver staining is recommended to detect minor contaminations that may still be present.
6. Use ultra-centrifugation filter units in order to concentrate the protein. Although not mandatory for FAHD proteins, in general a desalting step (e.g., by dialysis) is recommended for enzyme assays and crystallization.
7. Repeat steps 6.3–6.6 several times with different flow rates and salt concentrations (empirical) in order to enhance the purity of the FAHD protein. Wash the column overnight with H_2_O and 20% EtOH (in H_2_O).

### 7 Basic FAHD activity assays with substrates oxaloacetate and acetylpyruvate

**Figure 4:**
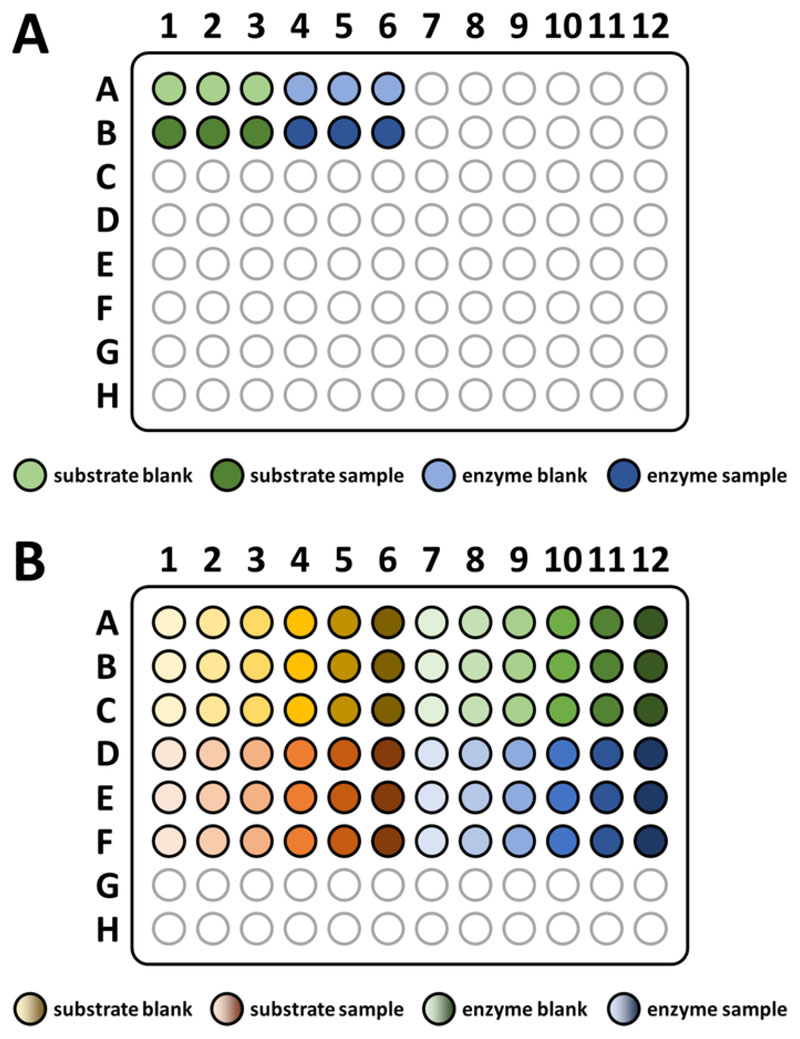
Sketched pipetting scheme for enzyme assays. **(A)** A sketched pipetting scheme for basic substrate-based FAHD protein enzyme assays. Substrate blank: -S/-E; substrate sample: +S/-E; enzyme blank: -S/+E; enzyme sample: +S/+E (S: substrate, E: enzyme). See protocol step 7 for more details. **(B)** A sketched pipetting scheme for assessing Michaelis-Menten kinetics of FAHD protein. Substrate blank: -S/-E; substrate sample: +S/-E; enzyme blank: -S/+E; enzyme sample: +S/+E (S: substrate, E: enzyme). See section 8 of the protocol for more details. Please click here to view a larger version of this figure.

NOTE: FAHD protein 1 (FAHD1) displays oxaloacetate decarboxylase (ODx) and acylpyruvate hydrolase (ApH) activity. This is outlined in more detail in the discussion section. Because of destabilization by keto-enol tautomerization in aqueous solution (i.e., enolization), oxaloacetate decays by itself over time (auto-decarboxylation) as a function of cofactor concentration and pH. At around a pH of 7 and temperature of 25 °C, this effect is not dramatic, but assays must be blanked to account for both auto-decarboxylation and enzyme concentration. The pipetting scheme is outlined in Figure 4A. In general, it is recommended to use well calibrated pipettes for this assay, as it is quite sensitive to minor pipetting errors.

**Figure 5:**
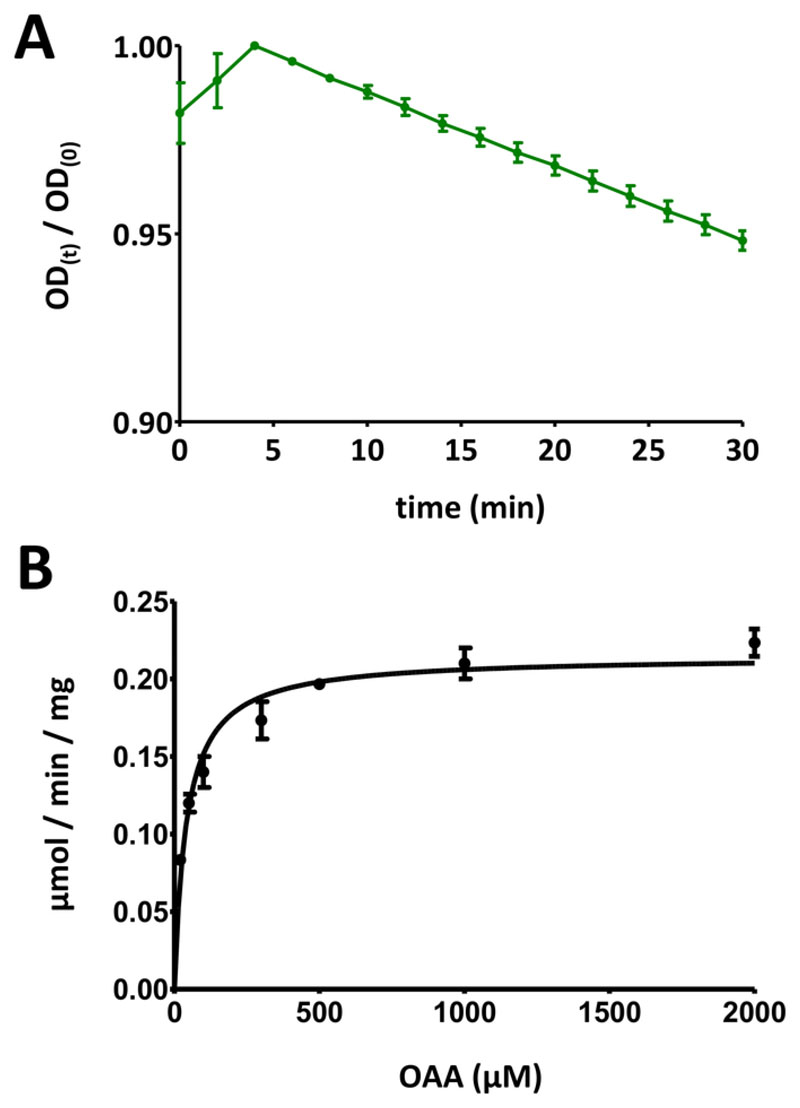
Exemplary results of enzyme assays. **(A)** An exemplary UV absorption curve obtained for basic substrate-based FAHD protein enzyme assays (normalized into the range of 0 to 1) with standard deviation. The optical density (OD) ratio [OD(t)/OD(0)] at any given time t [OD(t)] is normalized to the initial OD [t = 0; OD(0)]. See section 7 of the protocol for more details. **(B)** Exemplary Michaelis-Menten kinetics of the human FAHD1 protein with standard deviation. See section 8 of the protocol for more details. Please click here to view a larger version of this figure.

1. Start up a microplate reader and equilibrate for 30 min at 25 °C. Setup a program for reading 12 wells (as outlined in Figure 4A) at 255 nm. It is recommended to use 25 multiple readouts with 5 ms time delay. Setup a cycle to measure 15x every 2 min (30 min total).
2. By default, prepare an enzyme assay buffer (see Table of Materials) with 1 mM MgCl_2_ at pH 7.4. Variant FAHD proteins may require different cofactors or pH levels. Mg^2+^ and Mn^2+^ are known cofactors for FAHD1^3,11,12,21^.
3. Create a 1 μg/μL protein solution, diluting with enzyme assay buffer (Table of Materials).
4. Set up 1 mL of 20 mM solution of a substrate to be tested (so far identified substrates of FAHD proteins are listed elsewhere^3^) in enzyme assay buffer.
5. According to the pipetting scheme displayed in Figure 4A, prepare the enzyme blank and sample wells: pipet 90 μL of enzyme assay buffer (Table of Materials) into the wells with 5 μL (5 μg) of enzyme solution.
6. According to the pipetting scheme displayed in Figure 4A, prepare the substrate blank and sample wells: pipet 95 μL of enzyme assay buffer into the wells.
7. Right before measuring, apply 5 μL of enzyme assay buffer into the six blank wells. Apply 5 μL of the 20 mM substrate solution to the sample wells. It is recommended to use a multichannel pipette.
8. Use a multichannel pipette at 50 μL settings to gently mix all wells. Start with the blanks and proceed with the sample wells. Take care not to create any bubbles. Insert the plate into a microplate reader and measure each well at 255 nm (as outlined in step 7.1).
9. Perform the analysis in a spreadsheet. Copy the raw data from the photometer into a spreadsheet, and write all settings (i.e., all documentation) into another sheet. Average the data of the three wells of each of the four preparations. Subtract the blank from the sample. Also compute standard deviations and sum the deviations of blank and sample.
10. Plot this data (y: optical density, x: time in min). An exponentially decreasing curve should be displayed. Dependent on the kind of substrate in use, an initial increase within the first 10 min may be observed, after which the signal decreases. This is ascribed to the keto-enol tautomerization of the substrate, as outlined in more detail in the discussion section.
11. Divide the optical signal data over time by the maximum value of the plot, in order to scale the data down into the range [0, 1] (an example is provided in Figure 5A). Identify the linear range of the curve, starting at the initial decrease, and compute the negative slope (1/min).
12. The time course of the decrease in OD is associated to the substrate via its initial concentration: 100 nmol/well * slope. Using the assessed protein concentration c_0_, the specific activity is computed: 100 nmol/well * slope * 1/c_0_. Expressing c_0_ in μg/well, the specific activity computed this way is expressed using the unit nmol/min/μg, which equals μmol/min/mg.

### 8 Assessing Michaelis-Menten kinetics of FAHD proteins

NOTE: Assessing Michaelis-Menten kinetics of FAHD proteins is tedious, as the specific protein activity is dependent on both the relative protein-substrate concentration and physical volume in which the reaction is taking place. Steady-state kinetics must be established in order to obtain reliable results. A tested protocol on a 96 well UV transparent plate is outlined in the following steps. Every step needs to be performed with great care, as minor errors usually spoil the experiment. It is recommended to master the assays outlined in section 7 before attempting the more complicated assay described below.

1. Start a microplate reader and equilibrate for 30 min at 25 °C. Set up a program for reading 72 wells (as outlined in Figure 4B) at 255 nm. It is recommended to use 25 multiple readouts with a 5 ms time delay. Set up a cycle to measure 15x each 2 min (30 min total).
2. Perform steps 7.2 and 7.3. Then, set up 1 mL of 100 mM substrate solution in enzyme assay buffer.
3. Prepare dilutions of the substrate solution in enzyme assay buffer: 40 mM, 20 mM, 10 mM, 6 mM, 4 mM, 2 mM. The assay is performed with pairwise (“adjusted”) enzyme/substrate concentrations. For this, prepare the following dilutions of the enzyme solution in enzyme assay buffer: 0.5 μg/μL, 0.4 μg/μL, 2.5 μg/μL, 2 μg/μL, 1.5 μg/μL, 1 μg/μL.
4. Into all wells depicted in Figure 4B apply 180 μL of enzyme assay buffer. Apply 10 μL of enzyme assay buffer into all wells for the substrate (blank and sample). Apply 10 μL of the prepared protein dilution series into the wells for the enzyme (blank and sample). Apply 10 μL of enzyme assay buffer into all wells for wells for the substrate blank and the enzyme blank.
5. Right before measuring, apply 10 μL of the prepared substrate dilution series into the wells for the substrate sample and the enzyme sample.
6. Use a multichannel pipette at 50 μL settings to gently mix all wells, starting with the blanks, proceeding to the sample wells. Take care not to create any bubbles.
7. Insert the plate into a microplate reader and measure each well at 255 nm, as outlined in step 8.1. Perform the analysis in a spreadsheet. Copy the raw data from the photometer into a spreadsheet, write all settings (i.e., all documentation) into another sheet.
8. Perform individual data analysis per point in the dilution series as outlined in steps 7.11. to 7.14. Eventually, obtain all specific activities and plot against the initial substrate concentration: 2 mM, 1 mM, 0.5 mM, 0.3 mM, 0.2 mM, 0.1 mM.
9. Display all data points with individual standard deviations. Computer Michaelis-Menten kinetics *via* non-linear curve fitting, or via Lineweaver-Burk analysis. It may be required to re-measure individual points, and to adapt individual protein-concentration/substrate-concentration pair ratios in steps 8.5 and 8.6. The Michaelis-Menten diagram for human FAHD1 is provided in Figure 5B.

### 9 Crystallization of FAHD proteins

**Figure 6:**
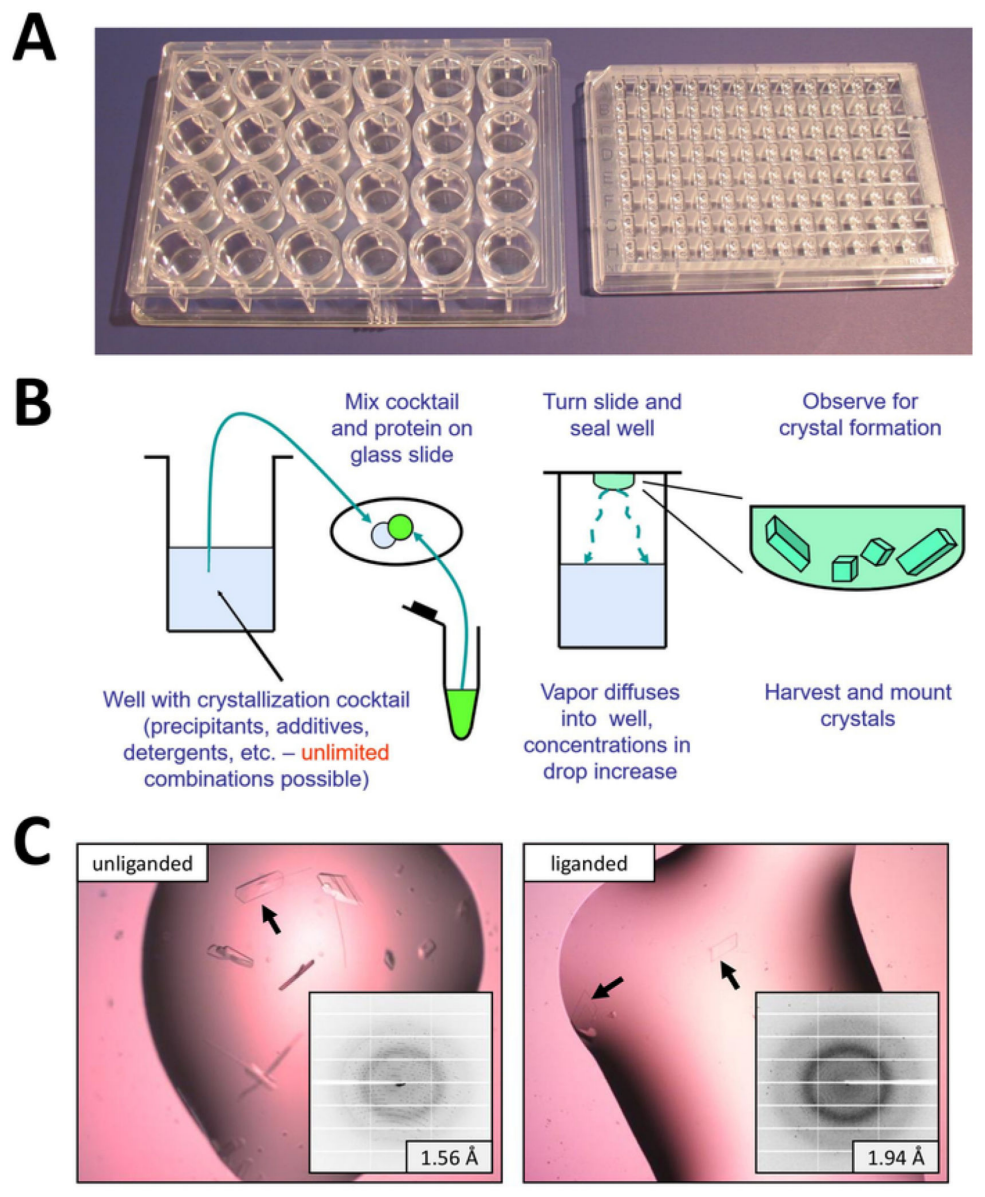
Crystallization of FAHD proteins. **(A)** Crystallization plates in standard 24 well or 96 well SBS footprint. See section 9 for more details. **(B)** The basic plate setup process in crystallization of FAHD proteins. This figure is redrawn with permission^23^. See section 9 for more details. **(C)** Human FAHD1 crystals and corresponding diffraction patterns (small inserts). The closest lattice spacing is indicated in the inserts as a measure for diffraction quality of the crystals. Lower numbers indicate higher resolution and thus more informative data. See section 9 of the protocol for more details. Please click here to view a larger version of this figure.

NOTE: Crystallization of FAHD proteins (human FAHD1 described previously^15^) may be achieved by the hanging drop vapor diffusion method in a 24 well format (Figure 6A). A step-by-step protocol on crystallization of human FAHD1 using this technique is presented below^15^. A more detailed description is provided in the discussion section.

1. Ensure that the protein is dialyzed against SEC running buffer. The FAHD1 protein should be available at high concentrations (2–5 mg/mL). At lower concentrations, the protein may not crystallize due to lack of spontaneous nucleation.
2. Prepare ≥20 mL of the reservoir solution for crystallization. Make three stock solutions, using distilled or deionized water as a solvent: 1 M Na-HEPES (minimum 25 mL, adjusted to pH 7.5), 50% (w/v) polyethylene glycol 4000 (PEG4k) (minimum 65 mL), and 1 M MgCl_2_ (10 mL).
3. Setup a grid of 4 x 6 (24 total) different 15 mL tubes. Label them according to corresponding positions on the plate (e.g., row (A, B, C, D) vs. column (1–6) like “A1”, “B5”, “D6”, etc.). Pipette 1 mL of 1 M Na-HEPES into each tube.
4. Pipette 1 mL of 50% (w/v) PEG4k into row A of the tubes, 2 mL into row B, 3 mL into row C, and 4 mL into row D. Pipette 100 μL of 1 M MgCl_2_ into column 1 of the tubes, 250 μL into column 2, 500 μL into column 3, 1.0 mL into column 4, 1.5 mL into column 5, and 2.0 mL into column 6.
5. Fill all tubes up to a 10 mL volume with distilled or deionized water, where the scale on the tubes is sufficiently accurate.
6. Take the human FAHD1 protein sample (~5 mg/mL) from the fridge (or from ice) and spin down at maximum speed with a table top centrifuge at 4 °C for at least 10 min. If co-crystallization with oxalate is desired, add oxalate from a stock solution so that the protein sample contains a final oxalate concentration of 2 mM. Apply 1 mM DTT and store on ice.
7. In the meantime, unpack a 24 well crystallization plate, ideally inside a temperature-controlled room at 18 °C. Distribute a thin layer of paraffin oil onto the rim on top of every well of the 24 well plate with the help of a thin glass or plastic rod. Add 800 μL of the prepared crystallization cocktails (A1 to D6) into each corresponding well of the crystallization plate.
8. Place fresh 22 mm coverslips onto a clean surface. Avoid contaminating the cover slips with dirt or dust. If necessary, remove any debris from the cover slip using compressed air or a duster spray.
9. After centrifugation is complete, avoid shaking the protein sample so that the spun-down aggregates and debris at the bottom of the tube do not float up again. In the following steps, pipette from the protein sample just below the surface of the solution in order to avoid stirring up aggregates and deposits from the bottom.
10. For each well (see Figure 6B) pipette 1 μL of protein solution onto the center of a cover slip and add 1 μL of the respective reservoir cocktail to the protein droplet, avoiding bubbles. Turn the coverslip upside down and place it onto the top of the well so that the oil seals the well with the coverslip air-tight. Repeat until the 24 well plate is completed.
11. Store the plate at 18 °C and observe the drops on a progressive schedule with a proper microscope. Human FAHD1 crystals usually appear overnight (see Figure 6C).

## Representative Results

**Figure 7:**
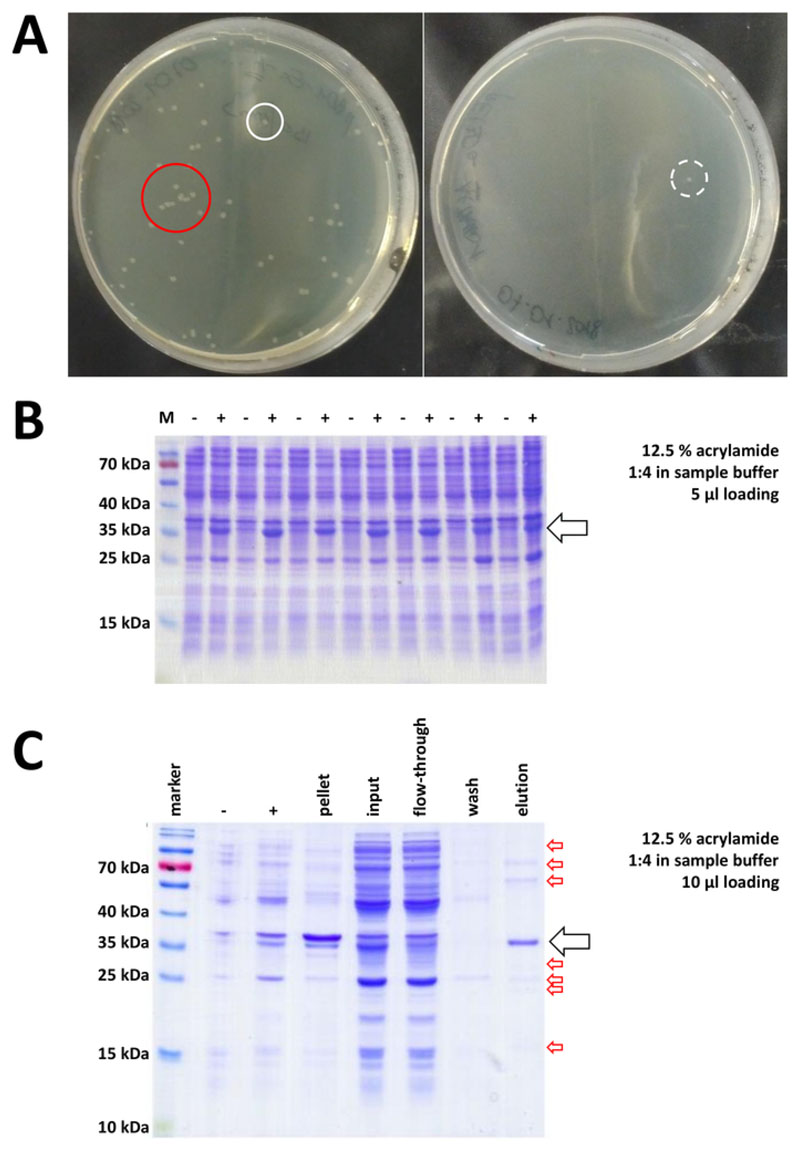
Representative results for bacteria transformation and IMAC. **(A)** Representative LB agar plates with transformed BL21(DE3) *E. coli*, obtained by following protocol step 1.1. Left: A plate with well distributed colonies (positive example). Right: A plate with only one single colony (negative example). White circles mark good colonies. The red circle marks colonies that are growing too close to each other and should not be picked as long as isolated colonies are available. **(B)** A 12.5% acrylamide SDS-PAGE analysis of a series of induction controls (“-“ indicates before IPTG induction; “+” indicates after IPTG induction, before pellet harvest), adjusted to equal amounts of total protein. This is described in step 1.2. **(C)** An exemplary 12.5% acrylamide SDS-PAGE analysis of Ni-NTA purification of *His*-tagged FAHD1 protein. This is described in section 3 of the protocol. The affinity chromatography yields protein of high purity (>70%, black arrow), however, several small contaminations are also observed (red arrows). These contaminations consist of non-FAHD proteins that bind to the column, and from proteins that bind to the FAHD protein. Please click here to view a larger version of this figure.

Starting with a prepared cloning vector and purchased BL21(DE3) *pLysS E. coli*, the plasmid is inserted into the bacteria via heat-shock or any appropriate alternative method (Figure 1). After a short period of amplification, the transformed bacteria are plated on LB agar plates, in order to grow overnight. Plates at this point may look different, depending on a variety of potential error sources. Plates may be empty (i.e., no colonies), completely overgrown by bacteria, or something in between, respectively. Two examples of LB agar plates after optimal and non-optimal transformation are depicted in Figure 7A. Too many bacterial colonies indicate either that too many bacteria were plated (likely) or that the antibiotics in use may be expired (unlikely). Too few bacterial colonies may indicate that either not enough plasmid was used for the transformation (use more next time) or that too much antibiotics were used to select the bacteria. In any case, if colonies are present, they should be fine, as using two selective antibiotics implies a rather insignificant chance of untransformed bacteria to grow. No colonies at all, however, indicates that either the bacteria lost their transformation competence (because of wrong storage or storage over longer periods, repetitive freeze and thaw, etc.), the heat-shock was not successful (no plasmid uptake or bacterial death by too much heat), the cloning vector is corrupted, or by mistake a wrong set of selective antibiotics was used (verify the resistance gene on the plasmid vector).

Validated colonies are selected and picked. After amplification in nourishing medium, protein expression is triggered by application of the chemical IPTG. The bacterial pellet containing the expressed protein in milligram quantities is harvested, and expression is verified via SDS-PAGE (see for example Figure 7B). Some problems may occur during this otherwise simple process. First, some proteins form inclusion bodies, because they apparently somehow interfere with the natural metabolism of the host bacteria. This was observed for some point mutations of human FAHD1 and FAHD2. In such cases, other expression systems like insect cells may be more appropriate and should be considered. After harvesting a pellet from insect cells, for example, purification of the proteins follows the same steps as described in this protocol. Second, the DE3-*pET* system is sometimes found to be “leaky” (i.e., protein is already expressed to some extent before IPTG induction). The potential reason for this is not well-understood, but it may help to express the protein slowly overnight in a cold room incubator. Third, no protein is expressed. This is probably the worst-case scenario, as it likely indicates a corrupted plasmid vector and thus advisable to sequence the plasmid.

**Figure 8:**
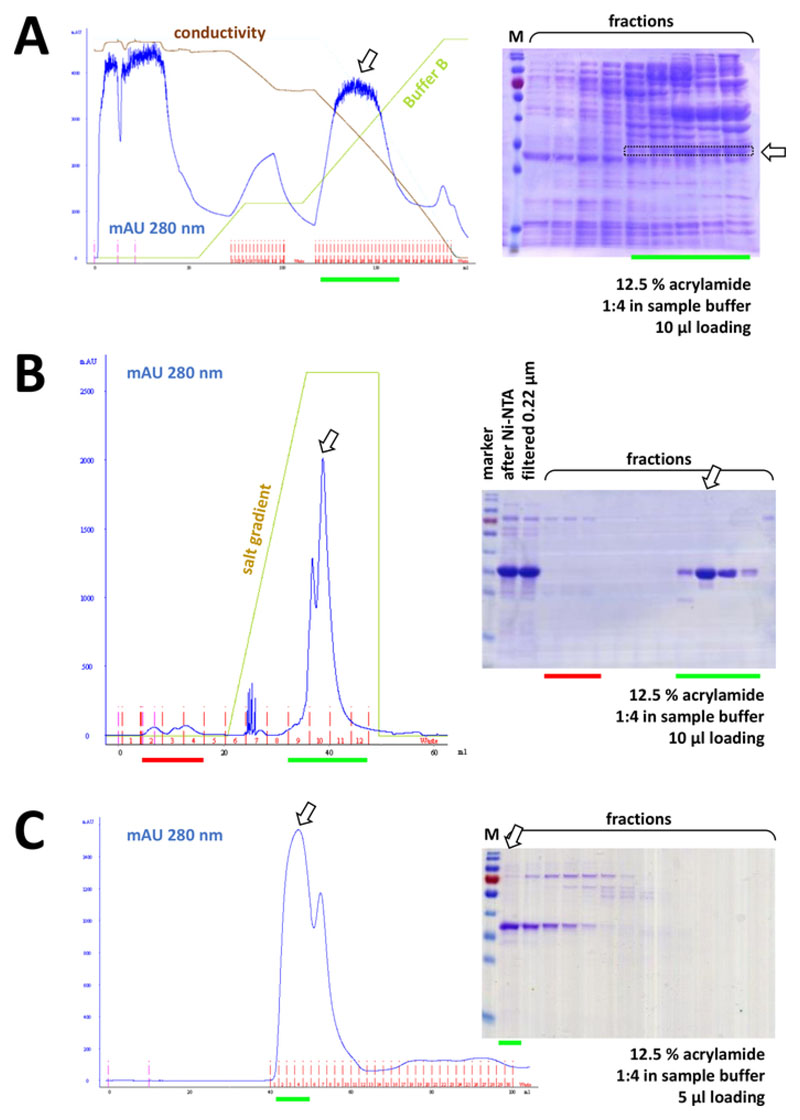
Representative results for FPLC experiments (HIC, ion exchange, SEC). **(A)** A typical chromatogram and 12.5% acrylamide SDS-PAGE analysis of HIC-phenyl chromatography after ammonium sulfate (AS) precipitation of untagged FAHD1 protein, as described in section 4 of the protocol. The green line reflects the gradient of buffer B that does not contain AS. During the process AS is gradually washed out from the system. Comparing this panel to Figure 7C displays the power of Ni-NTA affinity chromatography compared to the HIC-phenyl method, and the advantage of using a *His*-tag system for protein purification. **(B)** An exemplary chromatogram and 12.5% acrylamide SDS-PAGE analysis of cationic exchange chromatography of *His*-tagged FAHD following Ni-NTA purification. Using a salt gradient, the applied sample is separated into individual proteins. **(C)** An exemplary chromatogram and 12.5% acrylamide SDS-PAGE analysis of G75 size exclusion chromatography of *His*-tagged FAHD following cationic exchange chromatography. Please click here to view a larger version of this figure.

If a *His*-tag was used to tag the protein, affinity chromatography with Ni-NTA agarose is an easy and cheap capture method eliminating the majority of contaminations (Figure 7C). Similar methods exist for other tag systems (e.g., *STREP-II*). If no tag was used, a combination of ammonium sulfate precipitation and consecutive hydrophobic exchange chromatography may also separate the protein from the majority of other proteins (Figure 8A). However, comparing the two methods (Figure 7C vs. Figure 8A), the superiority of the Ni-NTA methods can be demonstrated by SDS-PAGE analysis. Using *His*-tagged protein is therefore advised.

Consecutively, the protein is further separated from leftover contaminations by cation/anion exchange chromatography (for example, see Figure 8B), followed by size-exclusion chromatography (for example, see Figure 8C). It is advised to set up an initial purification strategy in this order; however, these columns should be used in combination, subsequently and in variation, until the protein is sufficiently pure.

Simple activity assays, in order to test for “yes or no” decisions on active substrates and/or cofactors, may be performed with *His*-tagged proteins after Ni-NTA purification, or untagged proteins after the ionic exchange column. Specific activities and kinetic constants must be determined with protein of highest purity. Crystallization may be attempted with proteins after the ionic exchange column, but the quality of crystals almost always correlates with protein purity. Polyclonal antibodies may be raised against proteins at any stage of the purification protocol; however, here the quality also correlates with the protein purity.

## Discussion

### Critical Steps

FAHD proteins are very sensitive to salt concentrations. At low NaCl concentrations, the proteins may precipitate upon thawing, but they can usually be fully reconstituted at higher salt concentrations. That is, if a FAHD protein precipitates for some reason, it may be recovered or refolded with higher salt concentrations (>300 μM). Some more hydrophobic proteins, however, may not be recovered (for example, human FAHD2), but detergents such as CHAPS (maximum 1%) or glycerol (10%) may be used to keep them in stable solution. In any case, shock-freezing using liquid nitrogen and storage at -80 °C is recommended, as it is a gentle and slow process of thawing.

Some unexpected problems may occur during Ni-NTA purification in step 3.1.10. Of note, a higher OD in the second collected sample than in the first sample indicates a too high volume of the agarose resin (take a note and use less resin in the next experiment). Also, the agarose resin itself leads to an OD signal at 280 nm (i.e., disruption of the agarose resin bed will give artificial signals). In case of doubt, it is advised to use other methods like a Bradford or BSA assay to determine protein concentrations.

In enzymatic assays, there are three critical aspects to be considered. First, assessing the protein concentration is critical to obtain the correct specific activities. The level of purity of the protein is influencing the result and needs to be estimated. In case of tagged protein, the mass of the tag-part has to be computed, and the specific activity has to be correspondingly corrected. For simple assays described in section 7 of the protocol, Ni-NTA purity is sufficient to distinguish between active and inactive substrates, cofactors, etc. In the case of more complex Michaelis-Menten kinetics, all reactant and substrate concentrations must be correctly determined. Especially when using oxaloacetate (which auto-decarboxylates over time) the enzymatic part of the reaction must be corrected for auto-decarboxylation (under the assumption that both reactions occur simultaneously). Initial changes in the optical density signal addressed to keto-enol tautomerization of the substrate must be considered. Third, concentrations and volumes must be adjusted. A reaction with defined concentrations of enzyme and substrate may give different results dependent on the assay volume. If there is too much enzyme per well, adhesion of the liquid may in fact bias the result.

For assessing Michaelis-Menten kinetics it is recommended to perform initial experiments in 100 μL, 200 μL, and 300 μL batches in order to find the optimal combination. Similar aspects apply to the ratio of enzyme-substrate concentrations for kinetic assays. Too much enzyme per substrate or too much substrate per enzyme put the system outside the linear steady-state Michaelis range. Initial experiments are required to optimize these conditions. Exemplary adjustment for human FAHD1 (wild-type) protein are provided in section 8, resulting in kinetic diagrams (as presented in Figure 5B, for example).

For crystallization a droplet of protein solution is pipetted in the center of a coverslip and mixed with a droplet of crystallization cocktail, which is usually composed of a buffer (e.g., Tris-HCl, HEPES) and a precipitant (e.g., polyethylene glycol, ammonium sulfate). A droplet of inhibitor solution for co-crystallization (such as oxalate in this protocol) may optionally be applied. The coverslip is then placed upside down above a well of reservoir containing crystallization cocktail, sealing the well air tight with the help of sealant oil (Figure 6B). Ideally, no precipitation occurs within the drop at the beginning of the experiment meaning the protein remains in solution. Since precipitant concentration in the reservoir is higher than in the drop, the drop starts to lose water by evaporation into the atmosphere of the well until equilibrium with the reservoir is reached. The diffusion of water into the reservoir causes a slow volume decrease of the drop which in turn causes an increase of both, protein and precipitant concentration in the drop. If the protein solution reaches the required state of super-saturation and thus meta-stability, spontaneous nucleation followed by crystal growth can occur. Reaching the supersaturated state is a necessary but not sufficient condition for crystallization. Crystallization of proteins needs both, favorable thermodynamic and kinetic conditions, and heavily depends on the unpredictable properties of the protein to be crystallized^22^.

### Modifications and Troubleshooting

Expression of protein in *E. coli* may be inefficient. Varying IPTG concentrations, expression temperature, and amplification time, such as room temperature for several hours or in cold room overnight, may need to be tested for each new protein to find optimal conditions. Precipitation of protein in inclusion bodies is sometimes observed for more hydrophobic FAHD proteins. In such cases, protein expression in other model systems such as insect cells is recommended, as inclusion bodies are less likely to form^26^.

As FAHD proteins are sensitive to salt and cofactor concentrations, as well as pH, purification strategies for different homologues, orthologues, and point mutation variants may differ in individual settings. The purification methods described are developed for the wild-type human and mouse FAHD1 protein. Concentrations of chemicals, such as NaCl and imidazole, as well as pH, may have to be adapted for individual proteins with a different isoelectric point (pI). Also of note, not every *His*-tagged protein may bind well to a Ni-NTA resin. If protein binding to the Ni-NTA column is inefficient, adapted concentrations of NaCl and imidazole, as well as varying pH conditions in the Ni-NTA running buffer may help to improve the quality of the outcome. If not, skipping the Ni-NTA step and proceeding to the step of ionic exchange chromatography may also lead to a successful purification strategy. If a protein binds to the Ni-NTA column but cannot be eluted from the column, addition of some mM EDTA may help disrupt the Ni^2+^ complex.

Concerning the process of crystallization, it needs to be understood that self-organization of large and complex protein molecules into a regular periodic lattice is an inherently unlikely process that depends heavily on difficult to control kinetic parameters. Even small changes in the set-up used for crystallization can dramatically alter the result and no crystals will form. Protein purity is generally of paramount importance. As a rule of thumb, a heavily overloaded SDS-PAGE gel should not show other bands. Also, the sequence in which steps are performed may affect the outcome. As an example, to ensure reproducibility, it is often necessary to keep the pipetting sequence the same, then first add the protein, and finally add precipitant to the crystallization droplet (or vice versa). Whichever method used, it should be kept the same when trying to reproduce or scale-up experiments. If no crystals are observed following this protocol, the chemical precipitant composition, pH, drop size, and protein-to-precipitate ratio can be varied in small increments. Patience and consistent observations of the drops are of virtue.

### Remarks to Catalytic Mechanisms of FAHD1

**Figure 9:**
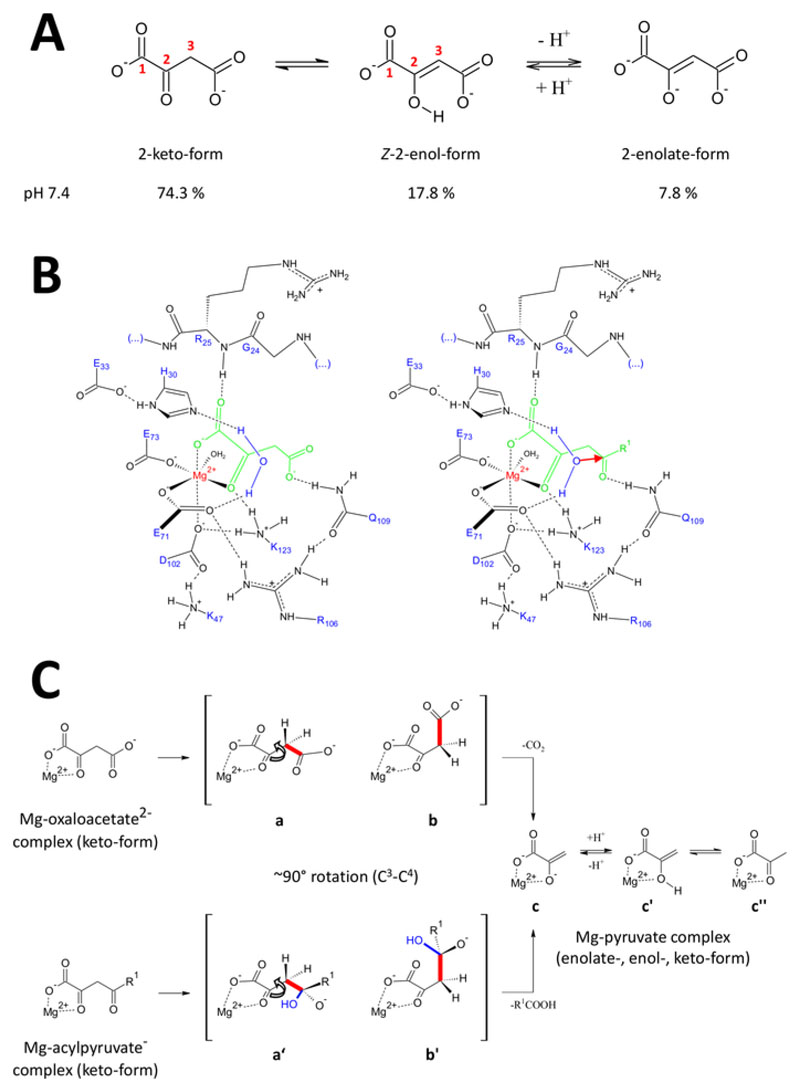
Details on the proposed catalytic mechanism of human FAHD1. **(A)** Oxaloacetate exists in crystalline state as well as in neutral solution mainly in the *Z*-enol form^24^. However, under physiological pH-conditions the 2-keto form is the predominant representation^25^. **(B)** Chemical sketch of the hFAHD1 cavity^15^ with Mg-bound oxaloacetate (left) and acylpyruvate (right, with R^1^ as organic rest; the red arrow denotes a nucleophilic attack of the adjacent stabilized water molecule) (see discussion). **(C)** Comparison of favored conformations for C^3^-C^4^ cleavage in decarboxylase (b to c) and hydrolase (b’ to c) mechanism of FAHD1: both processes result in Mg-complexed pyruvate-enolate (see discussion). Intermediates b and b’ are expected to be stabilized by Q109, as sketched in panel B (see discussion). Please click here to view a larger version of this figure.

The presented methods have been developed specifically to obtain FAHD1 proteins of high-quality. This enabled growth of FAHD1 crystals as well as engineering of crystals containing FAHD1 complexed to an inhibitor (oxalate, PDB:6FOG). The X-ray structures provide a 3D architecture of the enzyme’s catalytic cavity. These results establish a comprehensive description of residues potentially important for the catalytic mechanisms of this intriguing enzyme. FAHD1 was first described to be able to cleave acylpyruvates (acetylpyruvate, fumarylpyruvate)^11^. Later on, it was found that FAHD1 operates also as a decarboxylase of oxaloacetate^12^. Although the substrates acylpyruvate and oxaloacetate are different chemical moieties, the chemical transformations share mechanistically the strategic cleavage of a common single C^3^-C^4^ bond, energetically facilitated if the C^3^-C^4^ bond orbitals stay orthogonal to the π-orbitals of the C^2^-carbonyl^15^. Such a conformation allows resonance stabilization of the C^3^-carbanion transiently formed during the cleavage process. The FAHD1 substrates (oxaloacetate and acylpyruvates) are flexible molecules and may exist in tautomeric (keto-enol) as well as C^2^-hydrated forms (Figure 9A). The equilibria between the different species are determined mainly by the nature of buffer composition used, pH and presence of metal ions. In the following we discuss hypothetic mechanistic scenarios inspired from analysis of X-ray crystal structures which disclosed the catalytic center of FAHD1.

### The Decarboxylase Activity of FAHD1

Oxaloacetate exists in crystalline state as well as in neutral solution mainly in the *Z*-enol form^24^. But it was shown that under physiological pH-conditions (buffer conditions at pH 7.4) the 2-keto form is the predominant representation of oxaloacetate^25^ (Figure 9A), and that enolization is not a prerequisite for decarboxylation^27^. Of note, Mg^2+^ ions have no influence on the ratio of the oxaloacetate species at a pH of 7.4 or below^28^. Transposition of the oxaloacetate keto form into the catalytic center of FAHD1 (guided by the bound oxalate in the complexed enzyme (PDB: 6FOG^15^)) revealed residue Q109 as a conformational regulator of the bound oxaloacetate^15^. As outlined in another article^15^, hydrogen bonding to the carbamoyl group of Q109 stabilizes an oxaloacetate-conformation resulting from rotation around the C^2^-C^3^ bond (Figure 9B, left panel). As a consequence of this rotation, the C^3^-C^4^ bond (to be cleaved) adopts a close to orthogonal disposition relative to the π-orbitals of the C^2^-carbonyl (Figure 9C). Carbon dioxide can be released. The primary product of this process would be resonance stabilized Mg-enolate of pyruvate. It is known from investigations of oxaloacetate-Mg complexes that the enolate forms the most stable complex^28,29^. Assuming a comparable stability for a Mg-pyruvate enolate-complex the cofactor of FAHD1 could be blocked, but lysine residue K123 can protonate the pyruvate-enolate in an equilibrium to prohibit loss of the cofactor^15^.

The given interpretation suggests pyruvate enol as a distinct intermediate in the catalytic ODx function of FAHD1. At this step in the hypothesized model, experimental data does not provide any further indication as to why the closed lid should open to release the product. It may be deduced, however, that the proposed mechanism looks like an enzyme inhibition by the product: The crystal structure reveals a conserved water molecule held in directional orientation towards the FAHD1 catalytic center by residues H30 and E33 presented in a short helix^15^, which is induced upon ligand binding and lid closure. If the primary enol would stay in an equilibrium with the enolate, the resonance stabilized enolate could be quenched to pyruvate by the water molecule. The resulting hydroxyl would be capable to displace the pyruvate from the Mg-cofactor upon which the lid would open. Finally, the catalytic center would be restored in the mitochondrial environment. In this hypothetic scenario, the cavity water molecule would operate as an acid, respectively.

### Hydrolase Activity of FAHD1

Hydrolase activity of an enzyme implicitly requires the intermediate formation of a hydroxyl nucleophile. This mechanism is usually found in combination with acid-base catalytic activity. The transitional state of the reaction has to be prepared via conformational control by critical amino acid side chains in the cavity. In analogy to the discussion of the decarboxylase function, enzyme-bound acylpyruvate in 2-keto form will be put under conformational control by hydrogen-bonding of the 4-carbonyl oxygen to Q109 (Figure 9B, right panel). The crystal structure of oxalate-bound FAHD1 (PDB:6FOG) reveals a conserved water molecule held in directional orientation towards the FAHD1 catalytic center by residues H30 and E33 presented in a short helix^15^. The E33-H30 dyad is competent to deprotonate the directional positioned water and the resulting hydroxyl is in ideal disposition to attack the 4-carbonyl of acylpyruvate presented under conformational control by Q109^15^.

Of note, a similar mechanism has been proposed for FAH^18^. Attack by the hydroxyl nucleophile is expected to result in an oxyanion species, that is stabilized upon orbital controlled C^3^-C^4^ bond cleavage (Figure 9C). In this model, the C^3^-C^4^ bond rotation (Figure 9C) happens after the nucleophilic attack by the formed hydroxyl indicated in Figure 9B (i.e., it prepares the acylpyruvate for the bond cleavage). The primary products would be acetic acid and Mg-pyruvate enolate. In this hypothetic scenario, the acetic acid could quench the enol to pyruvate and subsequently assist displacement of the product. Above a pH of 7.5 and in the presence of Mg ions, acylpyruvates exist in an equilibrium between keto- and enol-forms, the latter in slight preference^30^. Most probably both forms are capable to bind to the cofactor of FAHD1 under subsequent lid closure. Processing of enolic acylpyruvate substrates by the enzyme is hampered due to the flat structure of the enol-form. The C^3^-C^4^ cleavage would result in a vinylic carbanion without resonance stabilization.

Therefore, we propose a catalytic ketonization step to prepare for attack of the hydroxyl nucleophile on the acyl carbonyl. This process of ketonization, however, would require control over proton transpositions by FAHD1 residues, which would attribute an inherent isomerase activity to FAHD1. It is reported that the acidity of Mg-bound enol hydrogen reveals a ten-thousand-fold increase compared to the un-complexed form^28^. A deprotonation of the Mg bound enol-form would be feasible by un-protonated K123. Deprotonation of K123 may be assisted by the carboxylate of D102. A hydrogen bond network formed by residues D102-K47-K123 could operate as the necessary proton relay in the catalytic center of FAHD1^15^. A such-formed intermediate enolate could then be quenched by a E33-H30-H_2_0 triad under ketonization of the substrate^15^. The 2-keto form would come under conformational control of Q109, and the concomitantly formed hydroxyl would attack the acyl carbonyl. The summarized discussion implies a control of FAHD1 about a water molecule for switching between acid and base through interplay of cavity-forming residues.

### Future Applications or Directions of the Method

Future applications of the methods described here are numerous. A plethora of prokaryotic members of the FAH superfamily still awaits functional characterization. Even the available information on the catalytic activities of known FAH superfamily members is scarce and, in most cases, based on theoretical assumptions rather than experimental data. Application of the methods described here for prokaryotic FAH superfamily members depends on the specific research interests in bacteriology. On the other hand, the recent demonstration that eukaryotic FAH superfamily members play essential roles in various cellular compartments (e.g., cytosol vs. mitochondria) highlights the need to better characterize these proteins (three of which have been identified so far), in particular because current data suggest that some uncharacterized proteins may carry out different functions in the context of mitochondrial biology, aging research, and cancer research. It is proposed that the full molecular and physiological characterization of these eukaryotic FAH superfamily members may provide important insight into major fields of contemporary research in the biomedical sector. More research on the mechanisms of FAHD1 (and related enzymes) are needed to better understand mechanisms underlying the bi-functionality of FAHD1, which is still not fully clarified. Additional studies with FAHD1 mutants, NMR-investigations, and structural studies on inhibitor complexes may help resolve the true mechanistic scenarios for which FAHD1 seems to be competent. Furthermore, computer-aided design of enol mimics capable to bind to the Mg-cofactor will eventually lead to potent inhibitors of FAHD1.

## Supplementary Material

Materials List
