# Supplementary material for: Expression, Purification, Crystallization, and Enzyme Assays of Fumarylacetoacetate Hydrolase Domain-Containing Proteins
Source: J Vis Exp. Author manuscript; Available in PMC 2020 Jul 30. (PMC7115867; doi:10.3791/59729)
Supplement: Materials List [file EMS85698-supplement-Materials_List.pdf]

Materials List for:

Expression, Purification, Crystallization, and Enzyme Assays of Fumarylacetoacetate Hydrolase Domain-Containing Proteins

Alexander K. H. Weiss[1,2], Max Holzknecht[1,2], Elia Cappuccio[1,2], Ilaria Dorigatti[1], Karin Kreidl[1], Andreas Naschberger[3], Bernhard Rupp[3], Hubert Gstach[4], Pidder Jansen-Dürr[1,2]

[1]Research Institute for Biomedical Aging Research, University of Innsbruck Austria
[2]Center for Molecular Biosciences Innsbruck (CMBI), University of Innsbruck Austria
[3]Division of Genetic Epidemiology, Medical University of Innsbruck Austria
[4]Faculty of Chemistry, Department of Organic Chemistry, University of Vienna Austria

Correspondence to: Alexander K. H. Weiss at alexander.weiss@uibk.ac.at

URL: https://www.jove.com/video/59729
DOI: doi:10.3791/59729

Materials

Name	Company	Catalog Number	Comments
BL21(DE3) pLysS competent E. coli	Promega	L1195	High-efficiency protein expression from gene with T7 promoter and ribosome binding site
pET E. coli T7 Expression Vectors	MERCK	-	http://www.merckmillipore.com/AT/de/life-science-research/genomic-analysis/dna-preparation-cloning/pet-expression-vectors/qFSb.qB.mLQAAAFA6.VkiQ0G,nav
0.45 µm filter units	MERCK	SLHP033NS	Millex-HP, 0.45 µm, PES 33 mm, not steril
0.22 µm filter units	MERCK	SLGP033RS	Millex-HP, 0.22 µm, PES 33 mm, not steril
Eppendof tubes 1.5 mL	VWR	525-1042	microcentrifugal tubes; autoclaved
15 mL Falcon	VWR	734-0451	centrifugal tubes
50 mL Falcon	VWR	734-0448	centrifugal tubes
PS Cuvettes Spectrophotometer Semi-Micro	VWR	30622-758	VIS transparent cuvettes
UV Cuvettes Spectrophotometer Semi-Micro	VWR	47727-024	UV/VIS transparent cuvettes
isopropyl-β-D-thiogalactopyranosid (IPTG)	ROTH	2316	chemical used for induction of protein expression with the DE3/pET system
imidazole	ROTH	X998	chemical used for elution of polyhistidine (6xHis) sequences from a nickel-charged affinity resin
Glass Econo-Column Columns	Bio-Rad	-	http://www.bio-rad.com/de-at/product/glass-econo-column-columns?ID=2cfb1c6e-32e8-4c72-b532-dd39013d707d&pcp_loc=catprod
chloramphenicol	Sigma-Aldrich	C0378	antibiotic for bacterial growth selection; resistance endióded in pLysS plasmid of BL21(DE3) E. coli; 25 µg/mL final concentration
kanamycin	Sigma-Aldrich	60615	antibiotic for bacterial growth selection; to be used if this resistance is encoded in the employed pET vector; 50 µg/mL final concentration

Copyright © 2019 Creative Commons Attribution-NonCommercial-NoDerivs 3.0 Unported License

ampicillin	Sigma-Aldrich	A1593	antibiotic for bacterial growth selection; to be used if this resistance is encoded in the employed pET vector; 100 µg/mL final concentration
Ultra-15, MWCO 10 kDa	Sigma-Aldrich	Z706345	centrifigal filters for protein enrichment; https://www.sigmaaldrich.com/catalog/product/sigma/z706345?lang=de®ion=AT
Ultra-0.5 Centrifugal Filter Units	Sigma-Aldrich	Z677108	centrifigal filters for protein enrichment; https://www.sigmaaldrich.com/catalog/product/ALDRICH/Z677108?lang=de&region=AT&cm_sp=Insite-_-prodRecCold_xviews-_-prodRecCold5-2
oxaloacetic acid	Sigma-Aldrich	O4126	TCA metabolite
sodium oxlalate	Sigma-Aldrich	71800	a competitive inhibitor of FAH superfamily enzymes
Dialysis tubing cellulose membrane	Sigma-Aldrich	D9277	https://www.sigmaaldrich.com/catalog/product/sigma/d9277; or comparable
Ni-NTA agarose	Thermo-Fischer	R90101	a nickel-charged affinity resin that can be used to purify recombinant proteins containing a polyhistidine (6xHis) sequence
96-Well UV Microplate	Thermo-Fischer	8404	UV/VIS transparent flat-bottom 96 well plates
PageRuler Prestained Protein Ladder, 10 to 180 kDa	Thermo-Fischer	26616	https://www.thermofisher.com/order/catalog/product/26616?SID=srch-hj-26616
ÄKTA FPLC system	GE Healthcare Life Sciences	-	using the FPLC system by GE Healthcare; different custom versions exist; this work used the "ÄKTA pure" system
HiTrap Phenyl HP column	GE Healthcare Life Sciences	-	https://www.gelifesciences.com/en/it/shop/chromatography/prepacked-columns/hydrophobic-interaction/hitrap-phenyl-hp-p-05630
Mono S 10/100 GL	GE Healthcare Life Sciences	-	https://www.gelifesciences.com/en/ch/shop/chromatography/prepacked-columns/ion-exchange/mono-s-cation-exchange-chromatography-column-p-00723
Mono Q 10/100 GL	GE Healthcare Life Sciences	-	https://www.gelifesciences.com/en/ch/shop/chromatography/prepacked-columns/ion-exchange/mono-q-anion-exchange-chromatography-column-p-00608
HiLoad Superdex column 75 pg (G75)	GE Healthcare Life Sciences	-	https://www.gelifesciences.com/en/ch/shop/chromatography/prepacked-columns/size-exclusion/hiload-superdex-75-pg-preparative-size-exclusion-chromatography-columns-p-05800
HiLoad Superdex column 200 pg (G200)	GE Healthcare Life Sciences	-	https://www.gelifesciences.com/en/ch/shop/chromatography/prepacked-columns/size-exclusion/hiload-superdex-200-

Copyright © 2019 Creative Commons Attribution-NonCommercial-NoDerivs 3.0 Unported License

			pg-preparative-size-exclusion-chromatography-columns-p-06283
TECAN microplate reader	TECAN Life Sciences	-	https://lifesciences.tecan.com/microplate-readers
acetylpyruvate	MoleculeCrafting.HuGs e.U.	-	custom synthesis
benzoylpyruvate	MoleculeCrafting.HuGs e.U.	-	custom synthesis
VDX™ plate (24 wells)	Hampton	HR3-142	24 well plates used for crystallization via Hanging Drop Vapor Diffusion
paraffin oil	Hampton	HR3-411	used for crystallization via Hanging Drop Vapor Diffusion
coverslips (22 mm)	Karl Hecht KG	14043	coverslips used for crystallization via Hanging Drop Vapor Diffusion
Luria broth (LB) medium	self-prepared	-	a general growth medium for E. coli: 5 g/L yeast extract; 10 g/L peptone from casein; 10 g/L sodium chloride; 12 g/L agar-agar
NZCYM medium	self-prepared	-	a better growth medium for E. coli, used for amplification: 10 g/L NZ amine; 5 g/L NaCl; 5 g/L yeast extract; 1 g/L casamino acids; 2 g/L MgSO4; adjust pH to 7.4
Luria broth (LB) agarose plates	self-prepared	-	autoclaved agarose plates containing LB-medium and antibiotics for bacterial groth selection; https://www.addgene.org/protocols/pouring-lb-agar-plates/
Ni-NTA running buffer	self-prepared	-	20 mM Tris-HCl pH 7,4; 50-300 mM NaCl; 10-200 mM imidazole; ranges: optimal value varies among FAHD proteins
Ni-NTA elution buffer	self-prepared	-	20 mM Tris-HCl pH 7,4; 50-300 mM NaCl; 200-500 mM imidazole; ranges: optimal value varies among FAHD proteins
HIC running buffer	self-prepared	-	44 mM NaH2PO4; 6 mM Na2HPO4; 100 mM NaCl; 20 mM DTT; adjust to pH 7
HIC running buffer AS	self-prepared	-	HIC running buffer saturated with ammonium sulfate (AS); adjust to pH 7: 70 g ammonium sulfate + 90 mL buffer, stirred overnight in the cold room; adjust to pH 7.0
Mono S low salt buffer	self-prepared	-	44 mM NaH2PO4; 6 mM Na2HPO4; 10-300 mM NaCl; ranges: optimal value varies among FAHD proteins
Mono S high salt buffer	self-prepared	-	44 mM NaH2PO4; 6 mM Na2HPO4; 1-2 M NaCl; ranges: optimal value varies among FAHD proteins
Mono Q low salt buffer	self-prepared	-	20 mM Tris-HCl; 15 mM NaCl; adjust to pH 8.0
Mono Q high salt buffer	self-prepared	-	20 mM Tris-HCl; 1 M NaCl; 10 % glycerol; adjust to pH 8.0
G75 / G200 running buffer	self-prepared	-	15 mM Tris-HCl; 300 mM NaCl; adjust to pH 7.4

Copyright © 2019 Creative Commons Attribution-NonCommercial-NoDerivs 3.0 Unported License

enzyme assay buffer	self-prepared	-	50 mM Tris-HCl pH7.4; 100 mM KCl; 1 mM $MgCl_2$
protein crystallization buffer	self-prepared	-	G75 / G200 running buffer with 1 mM DTT
reservoir solution for crystallization	self-prepared	-	100 mM Na-HEPES pH 7.5; 5-20 % (w/v) PEG4k; 10 mM-200 mM $MgCl_2$

Copyright © 2019 Creative Commons Attribution-NonCommercial-NoDerivs 3.0 Unported License
